# Modeling the Error of the Medtronic Paradigm Veo Enlite Glucose Sensor

**DOI:** 10.3390/s17061361

**Published:** 2017-06-12

**Authors:** Lyvia Biagi, Charrise M. Ramkissoon, Andrea Facchinetti, Yenny Leal, Josep Vehi

**Affiliations:** 1 Institut d’Informàtica i Aplicacions, Universitat de Girona, Campus de Montilivi, s/n, Edifici P4, 17071 Girona, Spain; lyvia.biagi@udg.edu (L.B.); charrise.ramkissoon@udg.edu (C.M.R.); 2 Federal University of Technology—Paraná, 85053-252 Guarapuava, Brazil; 3 Department of Information Engineering, University of Padova, 35131 Padova, Italy; facchine@dei.unipd.it; 4 Service of Diabetes, Endocrinology and Nutrition (UDEN), Institut d’Investigació Biomédica de Girona (IdIBGi), Avinguda de França s/n, 17007 Girona, Spain; yleal@idibgi.org; 5 CIBER Pathophysiology of Obesity and Nutrition, Instituto de Salud Carlos III, 28029 Madrid, Spain

**Keywords:** continuous glucose monitor, artificial pancreas, type 1 diabetes, sensor error, measurement noise, calibration error, enlite sensor

## Abstract

Continuous glucose monitors (CGMs) are prone to inaccuracy due to time lags, sensor drift, calibration errors, and measurement noise. The aim of this study is to derive the model of the error of the second generation Medtronic Paradigm Veo Enlite (ENL) sensor and compare it with the Dexcom SEVEN PLUS (7P), G4 PLATINUM (G4P), and advanced G4 for Artificial Pancreas studies (G4AP) systems. An enhanced methodology to a previously employed technique was utilized to dissect the sensor error into several components. The dataset used included 37 inpatient sessions in 10 subjects with type 1 diabetes (T1D), in which CGMs were worn in parallel and blood glucose (BG) samples were analyzed every 15 ± 5 min Calibration error and sensor drift of the ENL sensor was best described by a linear relationship related to the gain and offset. The mean time lag estimated by the model is 9.4 ± 6.5 min. The overall average mean absolute relative difference (MARD) of the ENL sensor was 11.68 ± 5.07% Calibration error had the highest contribution to total error in the ENL sensor. This was also reported in the 7P, G4P, and G4AP. The model of the ENL sensor error will be useful to test the in silico performance of CGM-based applications, i.e., the artificial pancreas, employing this kind of sensor.

## 1. Introduction

**Figure 1:**
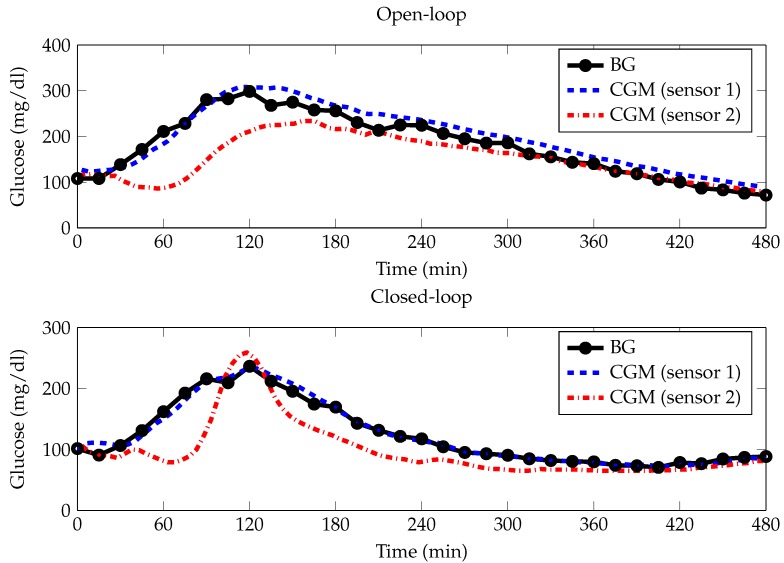
Representative example of the dataset employed in this paper. BG references frequently collected every 15 ± 5 min for 8 h (linearly interpolated by a straight line), and CGM time series (other lines) are measured simultaneously using *n* = 2 second generation Medtronic Paradigm Veo Enlite sensors (Northridge, CA, USA) in a type 1 diabetic patient during an open loop (top) and closed loop (bottom) meal test. BG, blood glucose; CGM, continuous glucose monitor.

The continuous glucose monitor (CGM) was initially used as a method for retrospective review of glucose profiles in those with type 1 diabetes (T1D). The first generations of CGMs approved by the Food and Drug Administration (FDA) beginning in 1999 were able to provide significant clinical benefits as an adjunct to standard self-monitoring of blood glucose (SMBG) [1,2,3,4]. Shortly after, real-time devices came about providing online glucose readings, but were widely acknowledged to have insufficient accuracy and reliability [5]. Nowadays, subsequent sensor generations are able to collect continuous (1–5 min sampling period) data for 7–14 days and can be used to determine glucose fluctuations. These new CGMs are recognized to be useful in the management of diabetes and can be used to improve glycemic control [6]. Despite these advantages, CGMs are still afflicted with errors related to accuracy, drift, time lags, calibration and noise, which affect the precision and accuracy of the blood glucose (BG) results [7,8,9]. Some of these errors are depicted in Figure 1, which shows a representation of the dataset used in this study. BG references were frequently collected every 15 ± 5 min for 8 h (linearly interpolated by a straight line), and CGM time series (other lines) are measured simultaneously using 2 second generation Medtronic Paradigm Veo Enlite (ENL) sensors (Northridge, CA, USA) in a T1D patient during a meal test. The top plot shows an example of an open-loop (OL) meal test with CGM (sensor 1) showing a slight continuous overestimation and CGM (sensor 2) showing a significant underestimation until time 210 min, likely due to a drift-in-time of sensor sensitivity. The bottom plot shows an example of a closed-loop (CL) meal test with CGM (sensor 2) displaying a time-lag at approximately time 43 min for about 1 h. All CGM time series exhibit random zero-mean measurement noise.

It is important to emphasize that most CGMs estimate BG from measurements of interstitial glucose (IG) [10]. The effects of calibration and blood-to-interstitium glucose (BG-IG) dynamics as potential confounders of the accuracy of CGMs are reported by [10,11]. As a result of recalibration and modeling of the IG dynamics, the authors found that the sensor accuracy is heavily dependent on the calibration procedures.

The availability of models of CGM sensor error is important for several CGM-based applications. First, the knowledge on the statistical nature of the error can be incorporated into CGM data signal processing algorithms to optimize their performance, e.g., to improve digital filters for denoising and calibration algorithms to reduce sensor inaccuracy. Second, a dissection of the error into its main components can give insight into which sources of error are prevalent in a specific sensor. Thirdly, it can be used to enhance the reliability of simulated CGM traces generated via e.g., the University of Virgina (UVA)/Padova T1D simulator [12]. Additionally, models of CGM sensor error can be used to improve the safety of an artificial pancreas (AP) system by allowing a controller to make more informed decisions based on known sensor error. As the efficacy of AP systems increase, more attention must be given to the overall safety of the system, with CGM error being a major contributor to the conservative approach to control in the AP. As knowledge of CGM errors grow, the ability to create and test more robust controllers increases. Finally, as recently seen with the Dexcom G5 Mobile sensor (San Diego, CA, USA), the availability of a detailed model of CGM sensor error allows specific sensors to be tested in silico [13] to determine if they can be safe and effective for non-adjunctive therapeutics decisions, i.e., insulin dosing [14,15].

In the past, several strategies to derive CGM sensor error characteristics have been proposed. Breton et al. [16] described sensor error using a first-order diffusion model and an autoregressive moving average (ARMA) model was considered to model the time dependency of consecutive errors. The authors analyzed two different datasets of the Abbott FreeStyle Navigator sensor (Chicago, IL, USA). A posteriori calibration was used to minimize the glucose discrepancy between sensor and reference. The authors noticed that the errors tend to be positive when the BG rate of change is negative and negative when the BG rate of change is positive, and that consecutive sensor errors are highly interdependent. Those procedures allowed the design of a simulator of sensor errors, but the model cannot describe errors due to calibration and sensor drift nor deal with interindividual variability of the BG-IG kinetics, although random fluctuations on CGM data were described in [17]. Laguna and colleagues [18] analyzed and modeled the error of the Dexcom SEVEN PLUS (7P; San Diego, CA, USA) and the first generation Medtronic Paradigm Veo Enlite sensors (Northridge, CA, USA) using a dataset of 12 subjects that wore the two sensors simultaneously. The sensor error was separated into lag time, the error stationarity, the error probability distribution, and the time correlation.

Recently, a technique to model the CGM sensor error from multiple simultaneous CGM traces has been developed by Facchinetti and colleagues [7,8] and it has been validated on CGM sensors of different generations produced by Dexcom Inc. (San Diego, CA, USA). In contrast to previous CGM error models, which cannot describe errors due to calibration and sensor drift nor deal with interindividual variability of the BG-IG kinetics. This method is innovative because it allows the sensor error to be dissected into its main key components, i.e., the delay due to the plasma-to-interstitium kinetics by assuming a physiological model of BG-IG kinetics, the calibration error that includes a model sensor sensitivity drift in time, and the measurement noise. In this paper, we apply a refined and enhanced version of the technique by Facchinetti and colleagues [7,8] to derive the sensor error model of the ENL sensor and we compare the results with those of the Dexcom 7P, the G4 PLATINUM (G4P)(San Diego, CA, USA), and the advanced G4 for artificial pancreas studies (G4AP) by Facchinetti and colleagues [7,8].

As discussed previously, the availability of the model of the ENL sensor error, which has not yet been derived, will be important to (i) simulate reliable CGM traces using T1D simulators, which will allow the pre-clinical in silico testing of CGM-based applications employing the ENL sensor, and (ii) test in silico if the ENL sensor is safe and effective for non-adjunctive use. Finally, this work will prove the reproducibility of the methodology proposed by Facchinetti and colleagues [7,8] to any sensor provided that suitable data is available.

## 2. Research Design and Methods

### 2.1. Study Population

Twenty patients with T1D were enrolled, ten at the Clinic University Hospital of Barcelona, Barcelona, Spain and ten at the Clinic University Hospital of Valencia, Valencia, Spain. The protocol was approved by the Ethics Committees of both hospitals (clinical settings). The selection criteria included the following: age between 18 and 60 years, duration of at least six months of continuous subcutaneous insulin infusion (CSII) therapy, body mass index (BMI) between 18 and 30 kg/m${}^{2}$, and HbA1c level between 6.0% and 8.5%. Patients on any experimental drug or use of an experimental device during the past 30 days were excluded. Patients with hypoglycemia unawareness, progressive fatal diseases, impaired hepatic or renal function, noncompliance, and/or, pregnant women were also excluded. See Table 1 for the demographic characteristics of the subject population.

### 2.2. Study Procedures

This was a randomized, prospective, one-way, repeated measures (four periods, two sequences) crossover study in subjects with T1D under continuous subcutaneous insulin infusion (CSII) [19,20]. Subjects underwent an 8-hour standardized mixed meal test (60 g carbohydrate, CH) on 4 occasions; on 2 occasions (CL1 and CL2), after a meal-announcement an augmented bolus was given, followed by manual adjustments of the basal rate every 15 min obtained via a CL controller; and on the other two occasions (OL1 and OL2), conventional CSII was used and boluses were based on the individual insulin-to-carbohydrate (I:CH) ratios. All subjects were randomly assigned to either sequence 1 (OL1-CL1-OL2-CL2) or 2 (CL1-OL1-CL2-OL2) with a wash-out period of at least 1 week between studies.

CSII was carried out with the Medtronic Paradigm Veo insulin pump (Northridge, CA, USA) and CGM using the second generation Medtronic Paradigm Veo Enlite sensors (Northridge, CA, USA). Two CGMs were inserted on either side of the umbilicus 24–48 h before the meal tests. In all subjects, calibration of CGM was performed according to the manufacturer’s instructions using the Contour Next Link (Ascensia Diabetes Care Holdings AG, Basel, Switzerland, Formerly Bayer). BG concentrations were measured every 15 ± 5 min with a YSI 2300 Stat Plus Glucose Analyzer (YSI 2300, YSI Incorporated Life Sciences, Yellow Springs, OH, USA).

### 2.3. Dataset

It is important to note that the trial was not designed to derive the error of the ENL sensor; the dataset was acquired for other purposes. The study was designed to analyze and compare the efficacy and safety of a newly developed CL algorithm implementing sliding mode reference conditioning (SMRC), adapted from a previous study [21], to current OL therapy during the postprandial period. It was only upon retrospective analysis that it was discovered that the data could be used to model the sensor error.

In total, 80 sessions were obtained; however, to be suitable for modeling, sensors must be inserted on the same day and only data in 10 patients fit this criteria. Therefore, 40 sessions were appropriate for the modeling approach used. From these 40 sessions, an additional three sessions were removed due to sensor malfunctions (i.e., signal loss). YSI data was interpolated via Bayesian smoothing [22] to one reading per minute and then aligned to CGM data to obtain a smooth BG profile.

### 2.4. CGM Error Model

**Figure 2:**
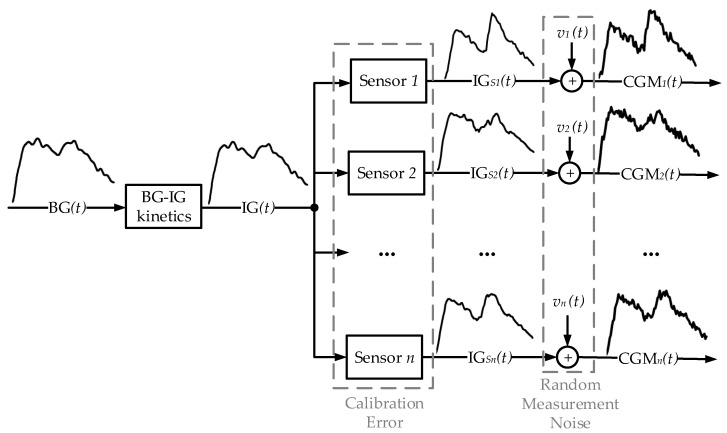
Description of how *n* parallel CGM signals are modeled. From left to right: transformation of the blood glucose (BG) signal into the interstitium glucose (IG) signal (BG-IG kinetics). Then, each of the *n* CGM sensors measures the IG signal, generating the IG${}_{Si}$ profile, which is susceptible to calibration error. Finally, the measured CGM${}_{i}$ is subject to random measurement noise, $v_{i}$.

The strategy employed to identify and model the sensor error is described in [7,8]. This approach was first used in [7] to model the 7P sensor error using 4 sensors in parallel (n=4). The authors then followed the same methodology in [8] to model the 7P, G4P, and G4AP sensors using 2 sensors in parallel (n=2). For completeness, all equations have been provided in this section. Figure 2 shows a schematic representation of CGM data streams (i=1,⋯,n) and the attribution of sensor error to the CGM output. The blocks represent the three components of the error: BG-IG kinetics, calibration error , and random measurement noise.

The IG concentration is converted into a BG concentration signal through BG-IG kinetics. The IG signal does not account for physiological variability due to perturbative influences such as physical activity and is therefore, assumed to be equivalent for all CGM channels in each subject. Each of the *i*-th sensors (i,⋯,n) measures the IG signal and generates the ${\mathrm{IG}}_{Si}$ profile.

Finally, the resultant BG output from each CGM sensor is affected by measurement noise $v_{i}\left(t\right)$:

(1) $${\mathrm{CGM}}_{i}\left(t\right)={\mathrm{IG}}_{Si}\left(t\right)+v_{i}\left(t\right).$$

#### 2.4.1. BG-IG Kinetics

The transformation of the BG signal to IG signal is modeled using the linear time-invariant two-compartment model, described in [23]. BG and IG concentrations are related by a convolution equation

(2) IG(t)=h(t)*BG(t),

where

(3) $$h\left(t\right)=\frac{1}{\tau }e^{-t/\tau }.$$

h(t) is the impulse response of the BG-IG system and τ is its respective time constant.

#### 2.4.2. Calibration Error

The relationship between IG and IG${}_{Si}$ is described as:

(4) $${\mathrm{IG}}_{Si}\left(t\right)=a_{i}\left(t\right)\mathrm{IG}\left(t\right)+b_{i}\left(t\right),$$

where $a_{i}\left(t\right)$ is time-varying gain and $b_{i}\left(t\right)$ is the offset, both specific to the *i*-th sensor.

Polynomial models are used to obtain a flexible description for gain ($a_{ik},k=0,\cdots ,m$) and offset ($b_{ik},k=0,\cdots ,l$) both specific to the i-th sensor, described as:

(5) $$a_{i}\left(t\right)=\sum_{k=0}^{m}a_{ik}t^{k},$$

(6) $$b_{i}\left(t\right)=\sum_{k=0}^{l}b_{ik}t^{k},$$

where *m* and *l* are the degrees of the polynomials and will be determined from the dataset.

#### 2.4.3. Measurement Noise

The zero-mean random measurement noise $v_{i}\left(t\right)$ that affects CGM${}_{i}$ signal is assumed to be composed by two signals: a common component cc(t) and a sensor specific component ssc${}_{i}\left(t\right)$:

(7) $$v_{i}\left(t\right)=\mathrm{cc}\left(t\right)+{\mathrm{ssc}}_{i}\left(t\right),$$

where cc(t) is common for all *n* residual profiles and ssc${}_{i}\left(t\right)$ is specific to the *i*-th sensor and uncorrelated with the other sensors. Both cc(t) and ssc${}_{i}\left(t\right)$ are modeled as autoregressive (AR) processes:

(8) $$\mathrm{cc}\left(t\right)=\sum_{k=1}^{r}\beta_{k}\mathrm{cc}\left(t-k\right)+w_{cc}\left(t\right),$$

(9) $${\mathrm{ssc}}_{i}\left(t\right)=\sum_{k=1}^{q}\alpha_{ik}{\mathrm{ssc}}_{i}\left(t-k\right)+w_{i}\left(t\right),$$

where *r* and *q* are the orders of the AR processes, $\left\{\beta_{k},k=0\cdots r\right\}$ and $\left\{\alpha_{ik},k=0\cdots q\right\}$ are the model parameters, and $w_{cc}\left(t\right)$ and $w_{i}\left(t\right)$ are zero-mean white noise processes.

### 2.5. Identification of the Unknown Parameters

First, the identification of the parameters of the submodels described in Equations (2), (5), and (6) was performed for all polynomial degrees of *m* (m=0,⋯,3) and *l* (l=0,⋯,3) in all combinations via nonlinear least squares. All estimations were done using MATLAB (Mathworks, Inc., Natwick, MA, USA). The use of nonlinear least squares simultaneously estimates all parameters at once to obtain a high goodness of fit; however, this approach may inherently produce undesirable parameters. To determine the precision of estimation, the coefficient of variation (CV) was calculated for each of the estimated parameters, and then the number of parameters that were estimated with elevated precision ((CV) < 20%) were counted.

Following the parameters estimation, the residual profile res${}_{i}\left(t\right)$ was then computed:

(10) $${\mathrm{res}}_{i}\left(t\right)={\mathrm{CGM}}_{i}\left(t\right)-\left(\sum_{k=0}^{m}{\hat{a}}_{ik}t^{k}\left(\frac{1}{\hat{\tau }}e^{-t/\hat{\tau }}*\mathrm{BG}\left(t\right)\right)+\sum_{k=0}^{l}{\hat{b}}_{ik}t^{k}\right),$$

where $\hat{\tau },{\hat{a}}_{i1},\cdots ,{\hat{a}}_{im},$ and ${\hat{b}}_{i1},\cdots ,{\hat{b}}_{il}$ are the outputs obtained for each combination of *m* and *l*. Except for $\hat{\tau }$, which is obtained for each individual, the other parameters represent a set of numerical values for each sensor and for each individual.

Next, the optimal order of the model was chosen by minimizing the Bayesian Information Criterion (BIC):

(11) $${\mathrm{BIC}}_{i}=d\ln\left({\mathrm{RSS}}_{i}\right)+p\ln\left(d\right),$$

where *d* is the total number of CGM data available in each sensor and each patient, p=(m+l+3) is the number of parameters, and RSS${}_{i}$ is the residual sum of squares as follows:

(12) $${\mathrm{RSS}}_{i}=\frac{1}{d_{i}}\sum_{j=1}^{d_{i}}\eta_{j}^{2},$$

where $d_{i}$ is the number of CGM samples of the *i*-th sensor and η is the uncorrelated version of measurement noise $v_{i}$. The BIC is a criterion for model selection among a set of models. The BIC index takes into account the statistical goodness of fit (first half of Equation (11)) and the number of parameters estimated (second half of Equation (11)), by imposing a penalty for increasing the number of parameters.

Next, to determine the optimal orders of *m* and *l*, ΔBIC was calculated as:

(13) $$\Delta \mathrm{BIC}={\mathrm{BIC}}_{\mathrm{LO}}-{\mathrm{BIC}}_{\mathrm{EHO}},$$

where ${\mathrm{BIC}}_{\mathrm{LO}}$ is the lower order BIC and ${\mathrm{BIC}}_{\mathrm{EHO}}$ is the equal or higher order BIC, determined by *p*. The advantages in the use of ΔBIC are: (i) it is more compact, requiring less boxplots to be plotted with no need to look at the absolute values of BIC and (ii) it is easier to read, where the distribution to the zero line is used to determine whether a more complex model should be used or not. In addition, comparing BIC values is equal to looking at the differences between the BIC for two selected models, i.e., the ΔBIC. The following steps were used to improve the choice of the orders *m* and *l* with respect to [7,8], where the orders were selected only by visual inspection of the ΔBIC boxplots. First, a *t*-test was performed on all combinations of ${\mathrm{BIC}}_{\mathrm{LO}}$ and ${\mathrm{BIC}}_{\mathrm{EHO}}$. For normally distributed data, p-values were calculated using the *ttest*, a parametric contrast technique ; all other data were analyzed using the Wilcoxon Ranksum test, a nonparametric contrast technique (α = 0.05 was considered to be the threshold of significance). The pairs of ${\mathrm{BIC}}_{\mathrm{LO}}$ and ${\mathrm{BIC}}_{\mathrm{EHO}}$ that obtained a statistically significant result were then ranked. The percentage of positive values and the mean of ΔBIC were used as determinants of model performance. The highest product between percentage of positive values and the mean of ΔBIC indicated the model with the highest performance.

After the optimal orders of *m* and *l* were determined, the realizations of common component, cc(t), and sensor specific component, ssc(t), were obtained. The availability of multiple sensors enables the decomposition of measurement noise into these two components described as follows:

(14) $$\hat{\mathrm{cc}}\left(t\right)=\frac{1}{n}\sum_{i=1}^{n}{\mathrm{res}}_{i}\left(t\right),$$

(15) $$\hat{{\mathrm{ssc}}_{i}}\left(t\right)={\mathrm{res}}_{i}\left(t\right)-\hat{\mathrm{cc}}\left(t\right).$$

$\hat{\mathrm{cc}}\left(t\right)$ and $\hat{{\mathrm{ssc}}_{i}}\left(t\right)$ were then modeled as AR processes of orders *r* and *q* (Equations (8) and (9)), which were determined by minimizing the calculated ${\mathrm{BIC}}_{\mathrm{AR}}$:

(16) $${\mathrm{BIC}}_{\mathrm{AR}}=n\ln\left(\mathrm{lss}\right)+k\ln\left(n\right),$$

where *n* is the length of the data (cc(t) or ssc${}_{i}\left(t\right)$), *k* is the order of the AR process, and lss is the loss function output of the MATLAB function ar. For each time series of cc(t) or ssc${}_{i}\left(t\right)$, ${\mathrm{BIC}}_{\mathrm{AR}}$ was calculated for all values of *k*, ranging from 1 to 15. The value of *k* that resulted in the lowest ${\mathrm{BIC}}_{\mathrm{AR}}$ was saved in a vector. The mode of these vectors, i.e., the orders that resulted in the minimum ${\mathrm{BIC}}_{\mathrm{AR}}$ most often, determined the optimal orders of the AR processes of both cc(t) or ssc${}_{i}\left(t\right)$. The goodness of the AR model of optimal order (AR(optimal order)) was tested using the Anderson–Darling test. These time series objects were then merged and the population AR processes for both common component and sensor specific component were identified using their previously determined optimal orders.

## 3. Results

**Figure 3:**
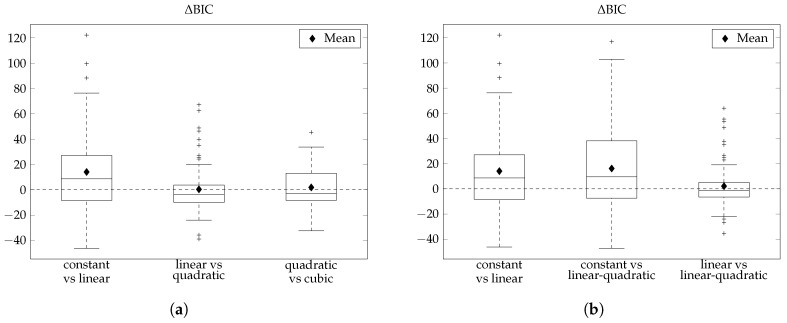
Boxplots of ΔBIC. (**a**) comparison between the non-hybrid models. (**b**) determination of the orders *m* and *l* of the polynomials $a_{i}\left(t\right)$ and $b_{i}\left(t\right)$. BIC, Bayesian information criterion.

The investigation of all combinations of *m* and *l* ranging from 0 to 3 produced intriguing hybrid results. Figure 3a depicts the boxplots of the ΔBIC between the non-hybrid models: constant versus linear (*p* = 0.0004), linear versus quadratic (*p* = 0.877) and quadratic versus cubic (*p* = 0.298). Ultimately, there were two models that we found to have the highest performances: the linear (*m* = *l* = 1) and the linear-quadratic (*m* = 1, *l* = 2). A summary of the results can be found in Figure 3b, where boxplots of the differences in BIC values between constant versus linear (left), constant versus linear-quadratic (middle) and linear versus linear-quadratic (right) are depicted.

The introduction of the linear term to the constant model as seen in the left boxplot has an average of 14.05 and is positive in about 62% of the cases (*p* = 0.0004), while the introduction of the linear term for *m* and quadratic term for *l* compared to the constant model is positive in 65% of the cases with an average of 16.14 (*p* = 0.0002). The right boxplot directly compares the linear model with the linear-quadratic model, which do not have a difference that is statistically significant (*p* = 0.317). The average of the right boxplot is 2.1 and is positive in about 46% of the cases.

Upon close inspection of the positivity, the mean, and the parameters estimated (see Table 2), it was found that the linear-quadratic model exhibited a performance that was only marginally higher than that of the linear model (*p* = 0.0002 vs. *p* = 0.0004) and the use of a more complex model could not be justified. Therefore, we chose to model the sensor error using the linear model because of its low complexity and high performance.

### 3.1. Parameters Estimated

To further compare the linear and linear-quadratic models, their parameters seen in Table 2 were analyzed. Table 2 reports the mean, standard deviation (SD), and percentage of values estimated with a CV < 20%. The use of CV allows us to ensure that all parameters were estimated with reasonable precision.

**Figure 4:**
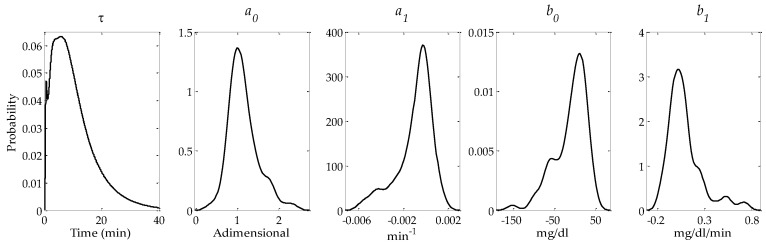
Probability density functions of parameters τ, $a_{0}$, $a_{1}$, $b_{0}$, and $b_{1}$ obtained applying a kernel density estimation procedure.

A *t*-test was performed between the common estimated parameters (τ, $a_{0}$, $a_{1}$, $b_{0}$, and $b_{1}$) of both models and no statistically significant difference was found (*p* = 0.052, *p* = 0.927, *p* = 0.882, *p* = 0.777, and *p* = 0.946, respectively). Looking at both the SD and the number of estimated parameters with a CV < 20%, it can be seen that the SD for the parameters estimated by the linear-quadratic model are larger, which infers a higher variability with no considerable increase in precision, reflected in the CV < 20%. These comparisons further indicate that the linear model is the model of choice. Figure 4 shows the distributions of τ, $a_{0}$, $a_{1}$, $b_{0}$, and $b_{1}$ obtained applying a kernel density estimation procedure for the optimal orders *m* = *l* = 1.

The mean and median of the estimated parameter of τ were 9.4 ± 6.5 min and 8.4 (5th = 0.3, 95th = 23.4) min, respectively. The gain parameters, $a_{0}$ and $a_{1}$, of the calibration error were estimated as 1.1 ± 0.4 min (median = 1.1 (5th = 0.7, 95th = 1.8)) and −0.0009 ± 0.0016 min (median = −0.0005 (5th = −0.0044, 95th = 0.0009), respectively. The offset parameters, $b_{0}$ and $b_{1}$, of the calibration error were estimated as −11.2 ± 38.8 mg/dL (median = −0.5 (5th = −90.7, 95th = 36.1)) and 0.09 ± 0.19 mg/dL/min (median = 0.05 (5th = −0.13, 95th = 0.52)), respectively.

### 3.2. Measurement Noise Level

Both cc(t) and ssc${}_{i}\left(t\right)$ have been modeled as realizations of AR processes. These signals can be optimally described by AR models of order 3, for cc(t) and order 2, for ssc${}_{i}\left(t\right)$. Table 3 reports the median variance of cc(t) and ssc${}_{i}\left(t\right)$ for the ENL (day 2), 7P (day 2) [7], G4P, and G4AP (day 4) [8] sensors. Regarding the variance of the processes, the variance of cc(t) is significantly greater than the variance of the ssc${}_{i}\left(t\right)$ (median values are 27.4 and 8.7 mg${}^{2}$/dL${}^{2}$, respectively, *p* < 0.0001 Wilcoxon Ranksum test).

The goodness of the identified AR model was validated by applying the Anderson–Darling test to the prediction-error times series e(t) (further information can be found in Facchinetti et al. [7]). It was found that 86.5% of the cc(t) time series and 67.6% of the ssc${}_{i}\left(t\right)$ time series passed the Anderson–Darling test, signifying that these processes through AR processes of order 3 and 2, respectively, are appropriate. The population AR processes for both cc(t) and ssc(t) are described as:

(17) $$\begin{array}{c} \mathrm{cc}\left(t\right)=1.584\;\mathrm{cc}\left(t-1\right)-0.8842\;\mathrm{cc}\left(t-2\right)+0.1798cc\left(t-3\right)+w_{cc}\left(t\right), \end{array}$$

where $w_{cc}\left(t\right)=N\left(0,3.98\;{\mathrm{mg}}^{2}/{\mathrm{dL}}^{2}\right)$ and

(18) ssc(t)=1.367ssc(t-1)-0.4816ssc(t-2)+w(t),

where $w\left(t\right)=N\left(0,2.54\;{\mathrm{mg}}^{2}/{\mathrm{dL}}^{2}\right)$.

### 3.3. Sensor Error Dissection

**Figure 5:**
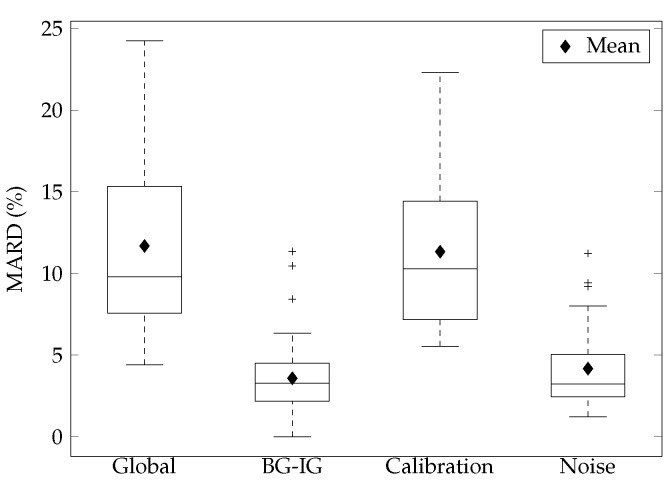
Boxplots of the mean absolute relative difference (MARD).

We used the mean absolute relative difference (MARD) to quantify the error components and overall error. The three key components of sensor error are the BG-IG diffusion process, calibration error (calibration), and measurement noise (noise). Figure 5 shows the CGM error of the ENL sensor obtained in this study. It should be noted that the sum of the components is greater than the global MARD because the global MARD does not take into account that the two signals are measured in two different compartments. Therefore, a bias exists allowing the error of one CGM to be canceled out by the accuracy of the other and vice versa. Table 4 includes our obtained values along with the values presented by Facchinetti and colleagues [7,8]. The global mean MARD was 11.7%, the MARD related to the BG-IG was 3.6%, the MARD related to calibration was 11.3%, and the MARD related to noise was 4.2%.

## 4. Discussion

This paper presents a sensor error model to represent the ENL sensor. This methodology dissects the sensor error into the delay due to the BG-IG kinetics, the calibration error, and the measurement noise. This is the first time that this methodology has been applied to the ENL sensor. The results are then compared to previously existing models of the 7P, G4P, and G4AP sensors, derived using the same methodology.

In Facchinetti et al. [7,8], *m* = *l* = 0 (constant), *m* = *l* = 1 (linear), *m* = *l* = 2 (quadratic), and *m* = *l* = 3 (cubic) were discussed in detail with a remark in Facchinetti et al. [7] stating that cases with m≠l were investigated but did not produce interesting results for their specific dataset. In contrast, when all combinations of *m* and *l* ranging from 0 to 3 were investigated in our dataset, we obtained intriguing non-hybrid and hybrid results. The boxplots of ΔBIC of the non-hybrid models exhibits more complex behavior, where the cubic model outperforms the quadratic model (Figure 3a), which was not seen in Facchinetti and colleagues [7,8]. As a result, the same methodology used by Facchinetti and colleagues [7,8], which relies on a visual inspection of the boxplots of the ΔBIC to choose model orders could not be implemented. Instead, an improved method that uses a statistical analysis of ΔBIC to determine the suitable model to represent the CGM error in the ENL sensor was employed. In the end, the linear model was chosen to model the ENL sensor.

The 7P sensor in Facchinetti et al. [7] was analyzed on day 2 after insertion and also obtained the optimal orders of *m* = *l* = 1, whereas, in Facchinetti et al. [8], the G4P and G4AP sensors were analyzed on days 1, 4 and 7 after insertion. Day 1 for both sensors obtained an optimal order of *m* = *l* = 1; however, on days 4 and 7, for both the G4P and G4AP, an optimal order of *m* = *l* = 0 was found. According to Facchinetti et al. [8], this lower order model found for days 4 and 7 is indicative that the time-variance of the calibration parameters of the G4P and G4AP sensors [24,25] tends to decrease during monitoring. The parameter values for the ENL sensor, the 7P sensor, and the G4P and G4AP sensors can be found in Table 2, Table 5, and Table 6, respectively.

The median value of τ reported in Facchinetti et al. [7] for the 7P sensor was 6.7 (5th = 2.2, 95th = 12.5) min. The mean values of τ reported in Facchinetti et al. [8] for the G4P and G4AP sensors were 9.7 ± 3.6 min and 7.7 ± 3 min, respectively. The τ of the ENL sensor has a similar average to that of the G4P but a higher amount of variability. The differences of τ seen between sensors can be attributed to greater delay variability, with respect to physiological variability and metabolic conditions [18], as well as varying sensor conditions, i.e., a larger range of CGM values in Christiansen et al. [24] and Garcia et al. [25]. Furthermore, the estimated τ for the G4P sensor is higher ( Table 6) than that reported in Keenan et al. (7.94 ± 6.48 min) [26], where the same sensor was analyzed. The variability observed in the time lag is well explained by the complexity of the plasma to interstitial glucose relationship [27].

The gain ($a_{0}$ and $a_{1}$) and the offset ($b_{0}$ and $b_{1}$) of the calibration error revealed that the G4P and G4AP sensors outperformed both the 7P and ENL sensors, while the $a_{0}$ parameter was estimated similarly for all sensors. The $a_{1}$, $b_{0}$, and $b_{1}$ parameters all had a higher mean and variation in the ENL sensor when compared to the G4P and G4AP sensors on day 1. The ENL and 7P sensors had similar median estimations for all parameters except $b_{0}$, where the 7P sensor exhibited a greater amount of error , possibly attributed to sensor drift and the accuracy of the estimated background current.

It has been observed that the CV found for the 7P, G4P and G4AP sensors are higher for the estimated parameters of $a_{1}$, $b_{0}$, and $b_{1}$. It is possible that the equation used to describe calibration error does not capture the behavior of the ENL sensor entirely and higher order models may be required as found by Laguna et al. [18] to explain the filtering and calibration algorithms used in this particular sensor.

In Facchinetti et al. [8], the authors report the median variance of cc(t) and ssc${}_{i}\left(t\right)$ for days 1, 4, and 7 of the G4P and the G4AP (Table 3). Day 1 infers that the sensor has been very recently inserted, while day 7 is near the end of the life of the sensor. Therefore, day 4 (standard working modality), which is not affected by the Foreign Body Response (FBR) or sensor hydration in the early stages of insertion [28,29] nor biofouling in the later stages of the sensor life [30], is the most appropriate day to compare to the ENL sensor on day 2. These values of variance are lower than those of the 7P, G4P, and G4AP sensors.

As reported in [7,8] we found that calibration error had the highest contribution to the global error observed. However, there was also a large number of outliers, showing that there may be higher variability in the error experienced by the ENL sensor than that of the 7P, G4P, and G4AP sensors. The BG-IG compares BG and IG time series, calibration compares IG with IG${}_{Si}$ and noise compares IG${}_{Si}$ and CGM${}_{i}$. Table 4 includes our obtained values along with the values presented in [7,8].

In [7], the mean MARD values for the 7P on day 2 are presented, while, in [8], the median MARD values of the 7P on day 1, and the G4P and G4AP on days 1, 4 and 7 are shown (Table 4). The comparison of the mean values between the ENL and 7P sensors [7] revealed a MARD reduction in not only the global analysis, but also in two of the three error components. The global mean MARD, the mean MARD related to calibration, and the mean MARD related to the noise of the ENL sensor all showed reductions when compared to the 7P, G4P, and G4AP sensors. The only error component that showed a higher value on the mean MARD was the BG-IG. The IG profile was obtained using the parameter τ and, as affirmed in [7], the estimation of τ is much more robust when multiple CGM sensors are present. Four CGM sensors in parallel (n=4) were used in [7], while, in our study and in [8], two sensors in parallel (n=2) were used.

The comparison of the median values between the ENL and the 7P, G4P, and G4AP sensors [8] showed a MARD reduction in the global analysis and in two of the three error components (Table 4). The global median MARD , the median MARD related to the noise, and the median MARD of the BG-IG of the ENL sensor all showed reductions when compared to the 7P, G4P, and G4AP. The median MARD related to calibration showed a reduction when compared to the 7P, but increased when compared to the G4P and G4AP. On average, all of the components showed a reduction of the median MARD of approximately 39% (7P), 9% (G4P) and 2% (G4AP). The global median MARD was reduced more than 30% (7P), 12% (G4P), and 2% (G4AP). In [8], only the day 1 MARD values were reported for the 7P, while the MARD data for days 1, 4 and 7 were aggregated for the G4P and G4AP. This aggregation of data amplifies the CGM error experienced, by combining the day of insertion (day 1) in which the signal is often unstable and more likely to be inaccurate [29] with the error near the end of the sensor life (day 7) [30].

**Figure 6:**
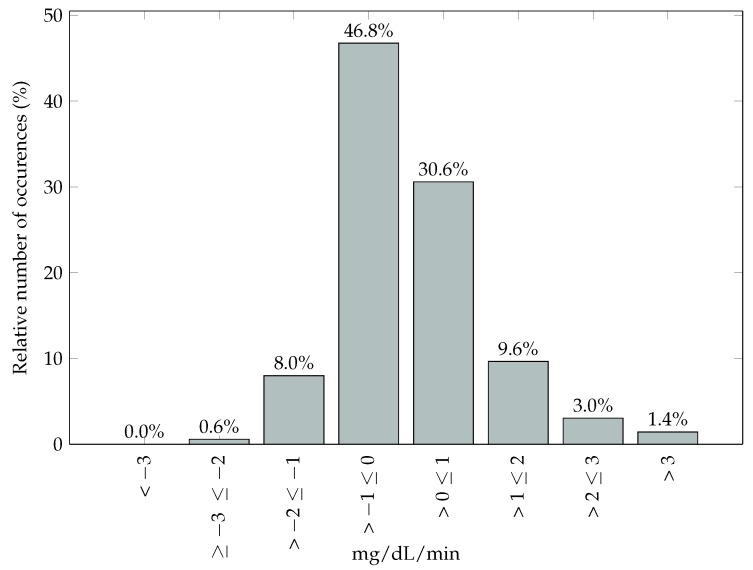
Glucose rate of change during trials.

A further comparison of the study protocol must be done to explain differences in the results we obtained compared to those of [7,8]. The protocol for Facchinetti et al. [7] can be found in Castle et al. [31], and, for Facchinetti et al. [8], the protocol can be found in Bailey et al. [32] (7P), Christiansen et al. [24] (G4P), and Garcia et al. [25] (G4AP). Table 7 presents the amount of subjects, inpatient sessions, duration of sessions, and the day at which the sensor was analyzed for all the studies compared in this paper. In this study and Castle el al. [31], the trials were done in a relatively controlled environment. In our CGM dataset, a rate of change between −1 and 1 mg/dL/min for 77.34% of the time was observed (Figure 6) and 97.76% of the values were in the euglycemic and hyperglycemic range (Table 8). While, in Bailey et al. [32], Christiansen et al. [24], and Garcia et al. [25], the meals, insulin doses, and meal timing were manipulated to obtain a full range of glucose values (from <60 mg/dL up to 400 mg/dL) during the in-clinic sessions.

This explains the higher MARDs presented in Facchinetti et al. [8], where it has been shown that, during rapidly changing conditions such as during a large meal or a hypoglycemic episode, CGM performance is poor [23,33,34,35,36]. It has been reported that, when compared, the G4P and ENL sensors exhibit a lower performance, especially in the hypoglycemic range [37]. To improve the comparison between the ENL, 7P, G4P, and G4AP sensors , datasets of the ENL and 7P sensors with similar conditions should be observed modeled. However, acquiring such a dataset is not easy and very expensive, since it requires an ad hoc trial and hospitalization for several hours in order to acquire frequent BG measurements in parallel to CGM data along with further safety measures for obtaining a full range of glucose values. At this point in time, this is the dataset available for the modeling of the error of the ENL sensor. With the availability of a dataset that observes a full range of glucose values as seen in [24,32], the ENL sensor error model and the comparison between sensors can be further improved.

Additionally, as seen in Table 7, 10 subjects were used in this study. In order to derive a solid model CGM sensor error, the dataset should be sufficiently large and represent the T1D population and its variability well. However, as stated above, acquiring such a dataset is difficult and very expensive. As previously mentioned and pointed out by Rossetti et al. [20], the dataset used in this paper was not acquired to derive CGM error model, but to compare CL and OL treatment during postprandial period. However, the dataset was suitable (even if not optimal) for the derivation of ENL sensor error into its different components and for comparing the resultant model with the previously decomposed error of other CGM sensors described in [7,8]. The ENL sensor error model could be refined with the availability of a larger dataset.

## 5. Conclusions

Not all CGMs are equal, and modeling and dissecting the error of various sensors allows us to understand the different errors that can be specific to a brand or model. In the present paper, we have modeled the ENL sensor, which is manufactured by Medtronic. The purpose of this paper was to apply an improved version of a previously presented CGM error modeling procedure [7,8], highlight any errors or sensor behavior that may be unique to the ENL sensor, and to compare sensor error in the ENL sensor to the error found in several sensors manufactured by Dexcom: the 7P, G4P, and G4AP.

The dissection of the sensor error into different components provided evidence that a large portion of CGM accuracy is related to the calibration, which was also reported by Facchinetti and colleagues [7,8]. The values of the mean and median MARD of the different components of the error showed a reduction in the majority of cases when compared to the values in Facchinetti and colleagues [7,8]. However, we must highlight that not only were the sensors that were analyzed different, there was also a large variation with regard to the day of sensor insertion and the protocol of the clinical trials conducted. The ENL sensor should be modeled using the same protocol found in Facchinetti et al. [8]. This will allow a more direct comparison of the ENL sensor to the 7P, G4P, and G4AP sensors Facchinetti et al. [8].

The models produced in this paper and by Facchinetti and colleagues [7,8] are intended to be used to create a sensor model bank that can be employed in a simulator to create more realistic simulations of real-life conditions and be used to enhance the performance of an AP system. Future works will compare the error created by the implementation of these models versus those of current simulators, which use white noise to create CGM error. Furthermore, additional models for each day of sensor life will be helpful in understanding the performance of the ENL sensor.

## Figures and Tables

**Table 1:** Demographic characteristics for the dataset used.

Number of T1D Patients	10
Sex	2 m, 8 f
Age (years)	44.5 ± 10.7
HbA1c (%)	7.9 ± 0.5
Body Mass Index (kg/m${}^{2}$)	27.6 ± 2.5
Time with T1D (years)	23.1 ± 9.7
Time with pump (years)	8.5 ± 4.4

Abbreviation: T1D, type 1 diabetes.

**Table 2:** Mean, standard deviation, 50th, 5th, and 95th percentile values for parameters generated considering models of order *m* = *l* = 1 and *m* = 1, *l* = 2 and percentage of values estimated with a coefficient of variation lower than 10%, 20% and 30% for the second generation Medtronic Paradigm Veo Enlite (ENL) sensor.

Model	Parameter	Mean	SD	Percentile			% of Values Estimate with		
				50th	5th	95th	CV < 10%	CV < 20%	CV < 30%
**ENL**	τ	9.4	6.5	8.4	0.3	23.4	92	95	95
***m*** **= 1**	$a_{0}$	1.1	0.4	1.1	0.7	1.8	100	100	100
***l*** **= 1**	$a_{1}$	-0.0009	0.0016	-0.0005	−0.0044	0.0009	74	85	91
***p*** **= 5**	$b_{0}$	−11.2	38.8	−0.5	−90.7	36.1	80	88	92
**day 2**	$b_{1}$	0.09	0.19	0.05	−0.13	0.52	70	81	86
	τ	10.1	7.0	9.3	1.5	24.3	97	97	97
**ENL**	$a_{0}$	1.1	0.4	1.1	0.6	1.8	100	100	100
***m*** **= 1**	$a_{1}$	−0.0010	0.0018	−0.0010	−0.0041	0.0030	77	89	91
***l*** **= 2**	$b_{0}$	−10.5	44.7	−9.0	−73.7	48.5	76	89	95
***p*** **= 6**	$b_{1}$	0.09	0.47	0.03	−0.31	0.78	72	84	92
**day 2**	$b_{2}$	0.0000	0.0008	0.0001	−0.0012	0.0008	72	82	89

Abbreviations: SD, standard deviation; CV, coefficient of variation.

**Table 3:** Median variance of cc(t) and ssc(t) for the second generation Medtronic Paradigm Veo Enlite (ENL) sensor and the Dexcom SEVEN PLUS (7P), G4 PLATINUM (G4P), and advanced G4 for artificial pancreas studies (G4AP) sensors.

	Day of Analysis	Median Variance	
		cc(t)	ssc(t)
**ENL**	2	27.4	8.7
**7P** ${}^{A}$	2	57.6	31.5
**G4P** ${}^{B}$	4	36.3	11.7
**G4AP** ${}^{B}$	4	31.0	8.9

${}^{A}$ Values reported in [7]; ${}^{B}$ Values reported in [8].

**Table 4:** Mean absolute relative difference (mean and median) values of the second generation Medtronic Paradigm Veo Enlite (ENL) sensor and the Dexcom SEVEN PLUS (7P), G4 PLATINUM (G4P), and advanced G4 for artificial pancreas studies (G4AP) sensors.

Sensor	Day of Analysis	MARD %				
			Global	BG-IG	Calibration	Noise
**ENL**	2	Mean	11.7	3.6	11.3	4.2
		Median	9.8	3.3	10.3	3.2
**7P** ${}^{A}$	2	Mean	14.2	3.5	12.8	5.6
**7P** ${}^{B}$	1	Median	14.1	6.8	14.1	5.4
**G4P** ${}^{B}$	1, 4, 7	Median	11.2	4.4	9.4	3.7
**G4AP** ${}^{B}$	1, 4, 7	Median	10.0	3.4	9.4	3.7

${}^{A}$ Values reported in [7]; ${}^{B}$ Values reported in [8]. Abbreviations: MARD, mean absolute relative difference; BG-IG, blood glucose to interstitium glucose.

**Table 5:** The 50th, 5th, and 95th percentile values for the parameters of the Dexcom SEVEN PLUS (7P) sensor and percentage of values estimated with a coefficient of variation lower than 10% and 30%.

Model	Parameter	Percentile			% of Values Estimate with	
		50th	5th	95th	CV < 10%	CV < 30%
	τ	6.7	2.2	12.5	97	97
**7P**	$a_{0}$	1.1	0.5	2.4	100	100
*m* = *l* = **1**	$a_{1}$	0.0002	-0.0044	0.0012	79	94
**day 2**	$b_{0}$	-14.8	-225.9	63.4	83	95
	$b_{1}$	0.04	-0.14	0.70	77	94

Values reported in [7]. Abbreviation: CV, coefficient of variation.

**Table 6:** Mean and standard deviation values for parameters and percentage of values estimated with a coefficient of variation lower than 20% for the Dexcom G4 PLATINUM (G4P) and advanced G4 for artificial pancreas studies (G4AP) sensors.

Model	Parameter	Mean	SD	% of Values Estimate with
				CV < 20%
	τ	9.7	3.6	–
**G4P**	$a_{0}$	1.16	0.31	100
*m* = *l* = **1**	$a_{1}$	-0.000116	0.000791	97
**day 1**	$b_{0}$	-9.4	55.6	97
	$b_{1}$	0.0027	0.1289	91
**G4P**	τ	9.7	3.6	–
*m* = *l* = **0**	$a_{0}$	1.04	0.16	100
**day 4**	$b_{0}$	2.8	15.8	100
**G4P**	τ	9.7	3.6	–
*m* = *l* = **0**	$a_{0}$	1.05	0.18	100
**day 7**	$b_{0}$	1.9	25.6	100
	τ	7.7	3.0	–
**G4AP**	$a_{0}$	1.09	0.26	100
*m* = *l* = **1**	$a_{1}$	-0.000060	0.000615	94
**day 1**	$b_{0}$	-6.4	50.1	94
	$b_{1}$	0.0133	0.1090	90
**G4AP**	τ	7.7	3.0	–
*m* = *l* = **0**	$a_{0}$	1.05	0.15	100
**day 4**	$b_{0}$	-2.6	14.9	100
**G4AP**	τ	7.7	3.0	–
*m* = *l* = **0**	$a_{0}$	1.07	0.13	100
**day 7**	$b_{0}$	-0.9	16.2	100

Values reported in [8]. Abbreviations: SD, standard deviation; CV, coefficient of variation.

**Table 7:** The total amount of subjects, in-clinic sessions, duration of each session, and number of days after sensor insertion for the datasets compared of the second generation Medtronic Paradigm Veo Enlite (ENL) sensor and the Dexcom SEVEN PLUS (7P), G4 PLATINUM (G4P), and advanced G4 for artificial pancreas studies (G4AP) sensors.

Sensor/Trial	Subjects	Sessions	Duration (h)	Sensor Day
**ENL**	10	37	8	2
**7P [31]**	19	36	9	2
**7P [32]**	53	53 *	8	1, 4, 7
**G4P [24] G4AP [25]**	36	108 *	12	1, 4, 7

* Not explicitly stated; calculated from known information.

**Table 8:** Samples separ ated per glycemic range. Time (%) and mean absolute relative difference (mean and standard deviation).

Glycemic Range (mg/dL)	*n*	Time	MARD	
		%	Mean	SD
**<70**	160	2.25	16.59	13.60
**70–180**	4584	64.45	12.60	10.84
**>180**	2369	33.31	9.57	9.08
**Overall**	7113	100	11.68	10.48

Abbreviations: MARD, mean absolute relative difference; SD, standard deviation.

## Abbreviations

The following abbreviations are used in this manuscript:

7P: SEVEN PLUS Sensor
AP: Artificial Pancreas
AR: Autoregressive
ARMA: Autoregressive Moving Average
BG: Blood Glucose
BG-IG: Blood-to-Interstitium Glucose Kinetics
BIC: Bayesian Information Criterion
BMI: Body Mass Index
CGM: Continuous Glucose Monitor
CH: Carbohydrate
CL: Closed-Loop
CSII: Continuous Subcutaneous Insulin Infusion
CV: Coefficient of Variation
ENL: Second Generation Medtronic Paradigm Veo Enlite Sensor
FDA: Food and Drug Administration
FBR: Foreign Body Response
G4AP: Advanced G4 Sensor for Artificial Pancreas Studies
G4P: G4 PLATINUM Sensor
IG: Interstitial Glucose
MARD: Mean Absolute Relative Difference
OL: Open-Loop
SD: Standard Deviation
SMBG: Self Monitoring Blood Glucose
SMRC: Sliding Mode Reference Conditioning
T1D: Type 1 Diabetes
